# Study of the Correlation Between Endophyte Abundances and Metabolite Levels in Different Parts of the Tissue of Cultivated and Wild *Arnebia euchroma* (Royle) Johnst. Based on Microbiome Analysis and Metabolomics

**DOI:** 10.3390/molecules30030734

**Published:** 2025-02-06

**Authors:** Jingjing Chen, Xiaoqing Zhang, Jinrong Zhao, Wenhuan Ding, Xuejia Zhang, Lan Pan, Haiyan Xu

**Affiliations:** 1 College of Traditional Chinese Medicine, Xinjiang Medical University, Urumqi 830017, China; 17799160495@163.com (J.C.); 15699091569@163.com (X.Z.); jinrongzhao957@163.com (J.Z.); dingding9089@sina.com (W.D.); xuejiazhang@126.com (X.Z.); panlan_sc@126.com (L.P.); 2 Central Laboratory, Xinjiang Medical University, Urumqi 830054, China; 3 Xinjiang Key Laboratory of Planting Standards for Authentic and Superior Chinese Medicinal Materials, Xinjiang Medical University, Urumqi 830017, China

**Keywords:** *Arnebia euchroma* (Royle) Johnst., endophytes, wild and cultivated, various tissues, diversity, chemical composition, multiomics, metabolomics

## Abstract

*Arnebia euchroma* (Royle) Johnst. has high medicinal and economic value, but in recent years, wild resources of this species have been depleted and the quality of artificially cultivated *A. euchroma* has been poor. The endophyte community of medicinal plants is rich, serving as an internal resource that promotes the growth of medicinal plants and the accumulation of secondary metabolites, and has important potential application value in improving the quality of medicinal materials. *A. euchroma* cultivars and wild varieties contain abundant endophyte communities and metabolites in different tissues. However, the relationships between *A. euchroma* endophytes and metabolites with different growth patterns and different tissue sites remain unclear. In this study, microbiome analysis and metabolomics were used to analyze the diversity of endophytes in the root and leaf tissues of cultivated and wild *A. euchroma* and their correlations with metabolites. The results revealed that the diversity of endophytes in *A. euchroma* was different from that in wild *A. euchroma* and that there was tissue specificity among different tissues. A species composition analysis revealed that the dominant endophytic fungi belonged to Ascomycota and Basidiomycota, and the dominant endophytic bacteria belonged to Proteobacteria and Cyanobacteria. A total of 248 metabolites, including quinones, flavonoids, alkaloids, organic acids, sugars, amino acids, coumarins, sterols, terpenoids, polyphenols, fatty ketones, and their derivatives, were identified in positive ion mode via LC–MS/MS. According to their different growth patterns and associated tissue parts, 9 differentially abundant metabolites were screened between AEZ-L (cultivated leaf tissue of *A. euchroma*) and AEY-L (wild leaf tissue of *A. euchroma*), 6 differentially abundant metabolites were screened between AEZ-R (cultivated root tissue of *A. euchroma*) and AEY-R (wild root tissue of *A. euchroma*), and 104 differentially abundant metabolites were screened between AEZ-R and AEZ-L. Eighty-two differentially abundant metabolites were screened between AEY-R and AEY-L. The contents of eight naphthoquinones in AEZ-R and AEY-R were determined via HPLC. The contents of *β,β’*-dimethylacrylylakanin in wild *A. euchroma* were greater than those in cultivated *A. euchroma*. A correlation analysis revealed that the dominant endophytes in the four groups were significantly correlated with a variety of metabolites, and the eight naphthoquinones in the root tissue were also significantly correlated with the dominant endophytes. The diversity of the *A. euchroma* endophyte community differed across different growth patterns and different tissue parts. There were significant differences in the relative contents of *A. euchroma* metabolites in different tissues. A correlation analysis verified the correlation between *A. euchroma* endophytes and metabolites.

## 1. Introduction

The radices of *Arnebia euchroma* (Royle) Johnst. and *Arnebia guttata* Bunge, plants belonging to the Boraginaceae family, are known as Arnebia Radix [1]. The dried root of *A. euchroma* is a high-quality product that is an important source of medicinal compounds. Arnebia Radix has many pharmacological effects and is often used in clinical applications for its wound healing, antibacterial, anti-inflammatory, antitumor, anticancer, blood lipid-lowering and liver protection properties. Different tissues of *A. euchroma* contain different types of compounds. Metabolomic studies have shown that *A. euchroma* contains naphthoquinones, terpenes, steroids, phenolic acids, alkaloids, polysaccharides, and other metabolites [2,3,4], among which the most representative constituents of *A. euchroma* in roots and their biological activities have been extensively studied. *A. euchroma* has high value in medicine, natural product recovery, food, the chemical industry and other fields [5,6], and the market demand for this species is increasing, leading to a short supply. However, *A. euchroma* has a narrow distribution, a small population, and weak reproductive ability, and its wild resources are on the verge of exhaustion [7]. At present, artificially cultivated *A. euchroma* has poor quality and substandard levels of effective components [8]. These factors, coupled with the harsh planting conditions, have restricted commercialization, leading to a failure to effectively alleviate the shortage of *A. euchroma* resources.

Endophytes are microorganisms, including bacteria, fungi, and actinomycetes, that live within healthy plant tissues or intercellular spaces for some of or all their lives without causing the host plant to show obvious symptoms of infection. Medicinal plant endophytes do not threaten biodiversity and can produce the same or similar bioactive metabolites as host plants. At present, endophytes are regarded as important sources of natural medicinal products. In recent years, numerous reports have demonstrated the close relationship between endophytes and the metabolites of medicinal plants. For example, endophytic fungi isolated from the medicinal plant *Coleus forskohlii* [9,10] can promote the synthesis and increase the content of trichothroxin, and the endophytic bacterium *Bacillus altitudinis,* isolated from *Panax ginseng* [11], can increase ginsenoside accumulation. Endophytic fungi isolated from *Taxus brevifolia* [12] can produce the medicinal compound paclitaxel, which has anticancer effects. Therefore, the endophytes of medicinal plants are abundant microbial resources involved in plant secondary metabolism, serving as alternatives to medicinal plants and alleviating the pressure caused by medicinal plant resource limitations.

Multiomic methods facilitate comprehensive scientific research through the integration of two or more omics techniques, contributing to a comprehensive and in-depth understanding of the complexity and dynamics of biological systems. In endophyte research for medicinal plants, joint analysis of the microbiome and metabolome has become a key approach, including not only systematic studies of the microbial community structure but also in-depth analyses of metabolite changes in medicinal plants, and is an effective strategy for studying the interactions between microorganisms and host plants. Joint multiomic analysis has been widely applied for in-depth research. For example, in *Panax quinquefolius* L. [13], the correlation between endophytes and differential metabolism was confirmed via microbiomics and metabolomics. The diversity of endophytes in *Rheum palmatum* from eight different producing areas in Gansu Province was compared via microbiome analysis and metabolomics, and the associations between these producing areas and five secondary metabolites (aloemodin, rhein, emodin, chrysophanol, and emodin methyl ether) were analyzed [14]. Therefore, a multiomic analysis of the microbiome and metabolome can reveal the pin-to-pin relationships among medicinal plants, microorganisms and metabolites, allowing for an exploration of their interactions, cross-verification, and a comprehensive analysis of the correlations between organisms.

At present, there are few reports on *A. euchroma* endophytes. JainR et al. [15] reported on the diversity of culturable endophytes of *A. euchroma* in the alpine desert region of the Himalayas from the perspective of ecological function. Endophytic flora play important roles in the adaptation of *A. euchroma* to abiotic stress and its acquisition of nutrients from dry and cold soils. Wang Yougui et al. [16] isolated the endophytic fungi of *A. euchroma*, fermented endophytic fungi of the genus *Penicillium*, and reported that the secondary metabolites of *Penicillium* had excellent broad-spectrum antibacterial activity. In addition, Zhang Jizhao et al. [17] explored the correlation between rhizosphere soil microorganisms of wild *A. euchroma* and the contents of medicinal components, providing guidance for the selection of ecological planting bases. However, in-depth research on *A. euchroma* endophytes has not been conducted, and the specific mechanism underlying the interaction between *A. euchroma* and endophytes remains unclear. In terms of metabolomics, Huang Rui et al. [8] studied six naphthoquinones in wild and cultivated Arnebia Radix and reported differences in the components of wild and cultivated Arnebia radix from three different locations. However, studies on the chemical components of *A. euchroma* have mainly focused on naphthoquinones in root tissue; the chemical components of other *A. euchroma* tissues have rarely been studied and these studies have not been comprehensive.

In this study, for the first time, the microbiome and metabolome were combined to analyze the relationships between cultivated and wild *A. euchroma,* as well as endophytes and *A. euchroma* in the roots and leaves of different tissues, and to explain the specific differences between different tissues and the quality differences between cultivated and wild *A. euchroma* from multiple perspectives. The main objectives of this study were as follows: (1) to analyze and compare the composition, diversity, and function of endophyte communities in the root and leaf tissues of cultivated and wild *A. euchroma* using high-throughput sequencing technology; (2) to investigate the distribution of metabolites in four *A. euchroma* samples, including AEZ-R, AEZ-L, AEY-R, and AEY-L, using ultra-high-performance liquid chromatography–mass spectrometry (UPLC–MS); (3) to determine the contents of eight naphthoquinones in AEZ-R and AEY-R using high-performance liquid chromatography; and (4) to analyze the correlations between the dominant endophyte communities and metabolites in the root and leaf tissues of cultivated and wild *A. euchroma* by combining microbiome analysis and metabolomics.

## 2. Results

### 2.1. Microbiome Study of Different Tissues (Root and Leaf Tissues) in Cultivated and Wild A. euchroma

#### 2.1.1. Results of the Surface Sterilization of *A. euchroma*

After 10 days of culture, there were no colonies on the nutrient agar (NA) medium and potato dextrose agar (PDA) medium in the control group, which indicated that the surface sterilization of the samples was effective and that the endophytes could be further studied.

#### 2.1.2. Determination of Endophyte Sequences Via High-Throughput Sequencing Technology

**Figure 1:**
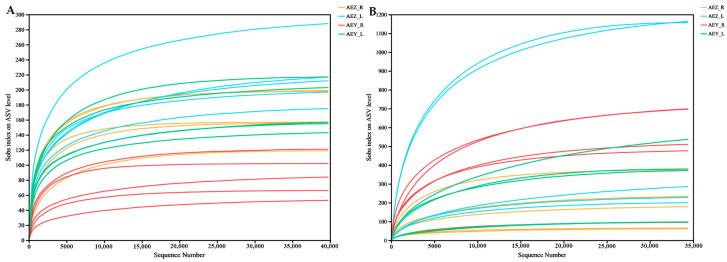
Rarefaction curves of endophytic fungi (**A**) and bacteria (**B**) in *A. euchroma* samples (AEZ-R1-5) (AEZ-L1-5) (AEY-R1-5) (AEY-L1-5). There were five biological replicates of cultivated roots, cultivated leaves, wild roots, and wild leaves.

Sequencing was carried out on AEZ-R, AEZ-L, AEY-R, and AEY-L (Table 1). A total of 1,311,218 valid sequences of endophytic fungi were obtained, with a sequence length of approximately 300 bp. The total number of valid sequences of endophytic bacteria was 937,621, with a sequence length of approximately 400 bp. Rarefaction curves (ASVs) can reflect the variations in species diversity and sample richness with sequencing quantity. With increasing sequencing volume, the sample rarefaction curve tended to be stable, indicating that the amount of sequencing data gradually became reasonable. As shown in Figure 1, all the curves tended to be flat, and the ASV coverage rate of all samples in each group was 99.8–99.9%, indicating that the amount of sequencing data was reasonable and allowed for complete coverage. Therefore, these data represent the true endophyte community structure of the sample, indicating that we could effectively analyze the endophyte community in each sample.

#### 2.1.3. Study of the Diversity of Endophytes in Different Tissues (Roots and Leaves) of Cultivated and Wild *A. euchroma*

**Figure 2:**
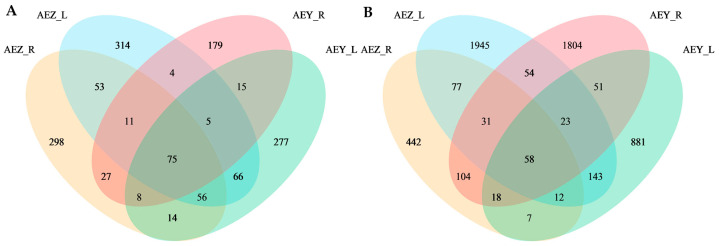
Venn diagrams of the ASV distributions of endophytic fungi (**A**) and bacteria (**B**) in the root and leaf tissues of cultivated and wild *A. euchroma.* The numbers in the figure represent the number of ASVs in each sample.

On the basis of the ASV classification level, a Venn diagram (Figure 2) was constructed, which revealed that the distribution of ASVs of endophytic fungi in the four groups of samples was as follows: in both wild and cultivated *A. euchroma*, the number of ASVs in leaf tissue was greater than that in root tissue; in both root and leaf tissues, there were more ASVs in cultivated *A. euchroma* than in the wild variety. Statistical analysis revealed that the number of ASVs shared by cultivated and wild *A. euchroma* and the number of ASVs shared by root and leaf tissues were low, indicating that the composition of endophytic fungi associated with different growth styles and tissues differed. The distribution of ASVs of endophytic bacteria in the four groups of samples was as follows: in cultivated *A. euchroma*, there were more ASVs in leaves than in roots; in the wild variety, there were more ASVs in roots than in leaves; in the root tissue, there were more ASVs in wild *A. euchroma* than in cultivated *A. euchroma*; and in the leaf tissue, there were more ASVs in cultivated *A. euchroma* than in wild *A. euchroma*. Statistical analysis revealed that cultivated and wild *A. euchroma* shared relatively few ASVs and that the root and leaf tissues shared relatively few ASVs, indicating that the composition of endophytic bacteria with different growth patterns and tissues also differed.

**Figure 3:**
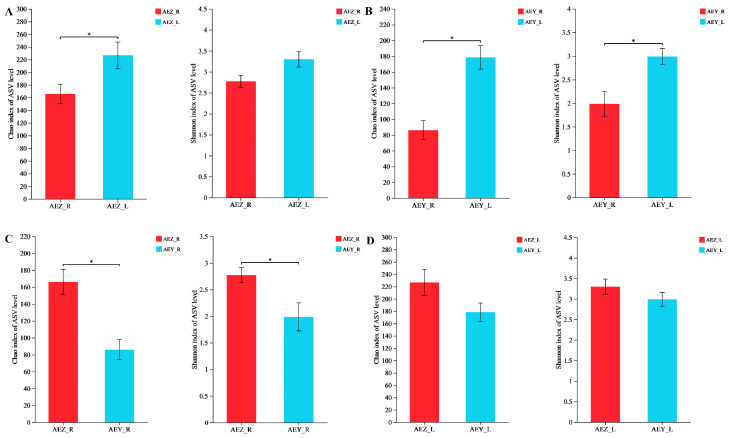
Differences in the Chao and Shannon indices between groups of *A. euchroma* endophytic fungi. (**A**) AEZ-R vs. AEZ-L; (**B**) AEY-R vs. AEY-L; (**C**) AEZ-R vs. AEY-R; (**D**) AEZ-L vs. AEY-L. * indicates a significant difference at the *p* < 0.05 level.

**Figure 4:**
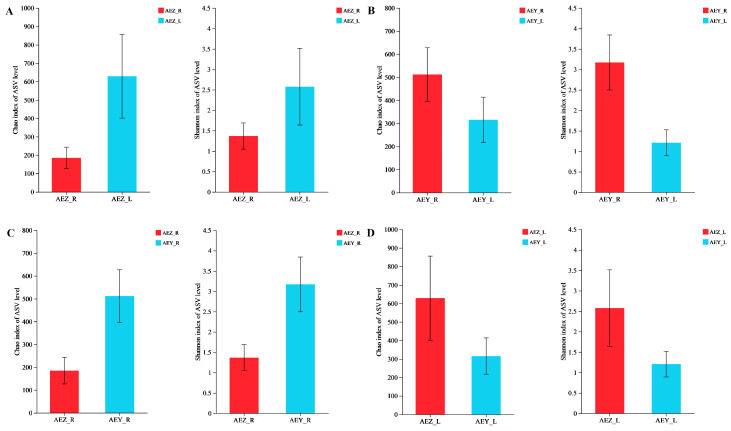
Intergroup differences in the Chao1 and Shannon indices of *A. euchroma* endophytic bacteria. (**A**) AEZ-R vs. AEZ-L; (**B**) AEY-R vs. AEY-L; (**C**) AEZ-R vs. AEY-R; (**D**) AEZ-L vs. AEY-L.

Diversity indices such as the Chao, Shannon, and coverage indices were calculated using mothur-1.30 software (Table 2). The Chao index reflects the abundance of endophytic flora in a sample, the Shannon index reflects the diversity of endophytic flora in a sample, and the coverage index reflects the community coverage. The closer the coverage value is to 1, the higher the coverage of the sample population, and the higher the probability of the sequence in the sample being measured. The table shows that the abundance and diversity of endophytic fungal flora in leaves were greater than those in roots and that those in cultivated *A. euchroma* were greater than those in wild *A. euchroma*. The endophytic bacterial diversity index (Chao) exhibited the following order: AEZ-L > AEY-R > AEY-L > AEZ-R. The Shannon index, reflecting the diversity of the flora, exhibited the following trends: cultivated *A. euchroma* > wild *A. euchroma*, leaf tissue > root tissue. Using R-3.3.1 software and the Wilcoxon rank sum test, the differences in the endophyte diversity index between different growth patterns and different tissues of *A. euchroma* were determined. In the endophytic fungal community, as shown in Figure 3, the Chao and Shannon indices of cultivated *A. euchroma* were greater than those of wild *A. euchroma* in different growth modes. Among the different tissues, both the Chao1 and Shannon indices were greater in the leaves than in the roots. When the two groups were compared, except for the nonsignificant difference in the Shannon index between AEZ-R and AEZ-L and the nonsignificant difference in the Chao index between AEZ-L and AEY-L, all the other comparisons revealed significant differences (*p* < 0.05). The results of the analysis of the endophytic bacterial community are shown in Figure 4. The Chao1 and Shannon indices of *A. euchroma* in different growth modes and different tissues were different, although not significantly so, and showed the following trends: AEZ-L > AEZ-R, AEY-R > AEY-L, AEY-R > AEZ-R, and AEZ-L > AEY-L. In general, the diversity of *A. euchroma* endophytes differed among different tissues and different growth modes, and the differences were more obvious in endophytic fungi than in endophytic bacteria.

**Figure 5:**
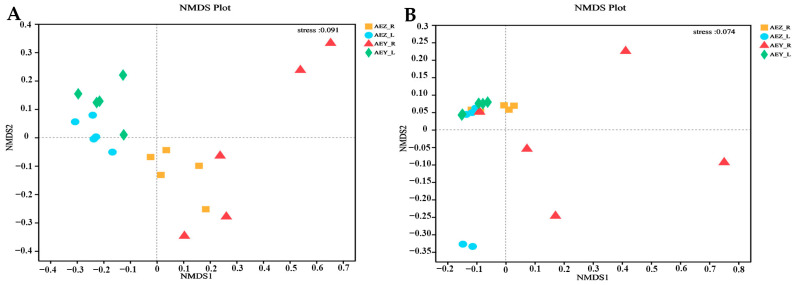
Multi-NMDS analysis of endophytic fungi (**A**) and bacteria (**B**). In the NMDS analysis results, points of different colors or shapes represent samples of different groups. The closer the two sample points are, the more similar the species composition of the two samples. The stress level < 0.2 indicates that the graph has some explanatory significance.

**Figure 6:**
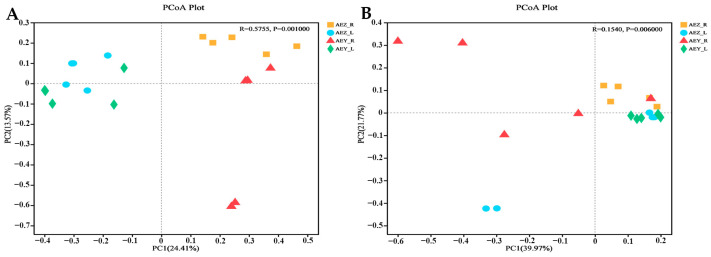
Principal coordinate analysis (PCoA) of endophytic fungi (**A**) and bacteria (**B**) in *A. euchroma* samples.

Beta diversity was analyzed on the basis of the ASV level, and the community structure composition of the endophytes in each sample was compared via NMDS (Non-metric Multidimensional Scaling) analysis in the ANOSIM (Analysis of Similarities) intergroup difference test, reflecting the differences between and within the sample groups. The analysis revealed a large difference in the endophyte community of cultivated *A. euchroma* and wild *A. euchroma* (Figure 5). There was a significant difference between root and leaf endophytes (*p* < 0.05). A principal coordinate analysis (PCoA) was performed using the ANOSIM intergroup difference test based on the Bray–Curtis distance algorithm. The results revealed that the total variation in endophytic fungi accounted for 24.41% of PC1 and 13.57% of PC2, and the total variation in endophytic bacteria accounted for 39.97% of PC1 and 21.77% of PC2. There was a certain degree of dispersion among the endophyte communities of the four groups, and the differences in endophytes among the samples were significant (*p* < 0.05) (Figure 6). There were significant differences in the composition of endophytic microbial communities in the root and leaf tissues of both cultivated and wild varieties. The compositions of the endophyte community in both leaf and root tissues were similar, but they differed between cultivated and wild varieties.

#### 2.1.4. Analysis of the Endophyte Community Composition in Different Tissues (Roots and Leaves) of Cultivated and Wild *A. euchroma*

**Figure 7:**
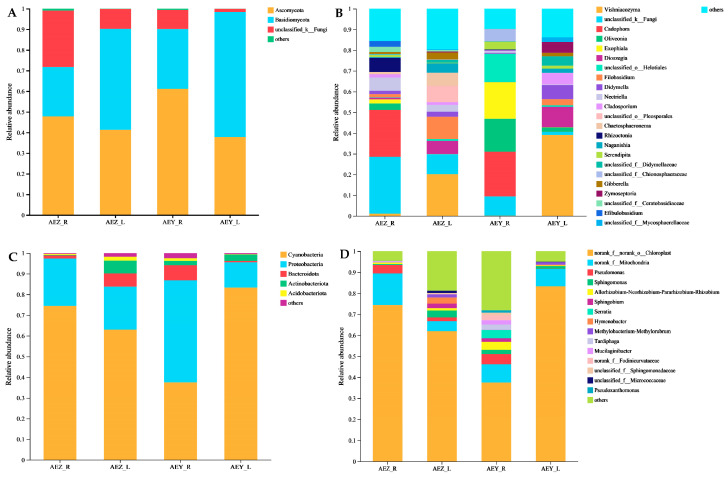
Composition of the relative abundances of endophytic fungi at the phylum (**A**) and genus (**B**) levels in *A. euchroma* samples. Relative abundances and composition of endophytic bacteria at the phylum (**C**) and genus (**D**) levels. The different colored columns represent different species, the lengths of the columns represent the proportion of the species, and other low-abundance species are classified as others.

A total of 1,311,218 effective sequences of endophytic fungi were obtained via high-throughput sequencing; these sequences were classified into 1402 ASVs, which were distributed in 12 phyla, 33 classes, 77 orders, 163 families, 314 genera, and 463 species. The 937,621 effective sequences of endophytic bacteria were classified into 1402 ASVs, which were distributed in 31 phyla, 86 classes, 200 orders, 324 families, 606 genera, and 1108 species. A bar map of the community at the phylum and genus levels was constructed (Figure 7). At the phylum level, Ascomycota was the most dominant phylum in AEZ-R and AEY-R, with relative abundances of 47.83% and 61.19%, respectively. The predominant phylum in AEZ-L and AEY-L was Basidiomycota, with relative abundances ranging from 48.95 to 60.65%. Endemic flora in AEZ-R, AEZ-L, AEY-R, and AEY-L were not identified at the phylum level. At the phylum level, Proteobacteria was the most dominant phylum in AEY-R, and its relative abundance was 49.26%. The dominant bacteria in AEZ-R, AEZ-L and AEY-L were Cyanobacteria, with relative abundances ranging from 63.05 to 83.35%. Endemic flora in AEZ-R, AEZ-L, AEY-R, and AEY-L were not identified at the phylum level. In general, there are many differences in the community composition of the four groups of samples at the phylum level.

At the genus level, Vishniacozyma was the most dominant genus of endophytic fungi in AEZ-L and AEY-L, with relative abundances of 14.24% and 34.98%, respectively. The predominant strain in AEZ-R was unclassified-k-Fungi, with a relative abundance of 26.88%. The predominant strains in AEY-R were *Cadophora* sp., Oliveonia pauxilla, unclassified o-Helotiales, and Exophiala angulospora, with relative abundances of 16.75%, 15.86%, 13.59%, and 14.43%, respectively. The predominant strains in cultivated *A. euchroma* were *Ceratobasidiaceae* sp., unclassified-g-Gibberella, and Chaetosphaeronema hispidulum. The genera endemic to leaf tissue were unclassified-f-Mycosphaerellaceae and Dioszegia fristingensis. The genera endemic to root tissue were *Serendipita* spp., Cadophora orchidicola, Rhizoctonia fusispora, *Cadophora* spp., and Cadophora orchidicola. In AEZ-R, the endemic strains were *Efibulobasidium* sp. and Cadophora luteoolivacea; in AEZ-L, the endemic strain was unclassified o-Pleosporales; in AEY-R, the endemic strains were Exophiala angulospora and *Chionosphaereaceae* sp.; in AEY-L, the endemic strain was Zymoseptoria versleyi. It can be concluded that, at the genus level, both cultivated and wild endophytic fungi have more endophyte species in the leaf tissues than in root tissues. Compared with wild *A. euchroma*, cultivated *A. euchroma* had more diverse flora in both the root and leaf tissues. The predominant bacteria in AEZ-R, AEZ-L, AEY-R, and AEY-L were norank_f_norank_o_chloroplasts, and their relative abundances ranged from 37.49 to 83.33%. Tardiphaga and Mucilaginibacter were characteristic of root tissue. In leaf tissue, the endemic strains belonged to Hymenobacter. In AEY-R, the endemic strains were Pseudoxanthomonas, Pseudomonas, Serratia, and norank_f_Fodinicurvataceae. The compositions of endophytic bacteria in different tissues differed at the genus level. In terms of relative abundance, AEY-R was unique and exhibited some differences to the other three groups.

**Figure 8:**
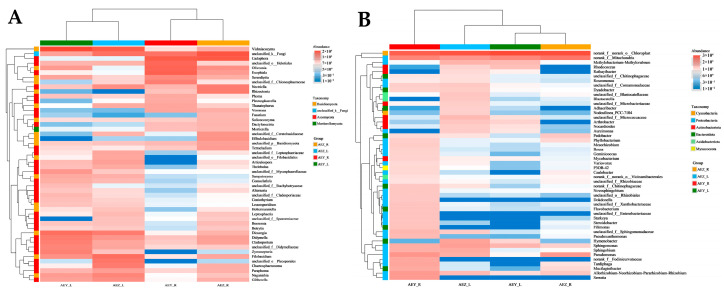
Heatmaps of the endophytic fungal (**A**) and bacterial (**B**) communities of *A. euchroma* samples at the genus level.

To better understand the differences in the community structure and abundance of endophytes in the tissues of cultivated and wild *A. euchroma*, the 50 genera with the highest abundance were selected, and a species clustering heatmap was generated (Figure 8). The relative abundance of species is indicated by the depth of the color, with red indicating high species abundance and blue indicating low species abundance. In the figure, the four groups of samples were clustered into two groups at the genus level according to the distribution and abundance characteristics of the fungi: the first group consisted of AEZ-R and AEY-R, and the second group consisted of AEZ-L and AEY-L. This once again verified the differences in the composition of endophytic fungal communities in roots and leaves between different tissues in both cultivated and wild *A. euchroma*. The differences in the community structure and abundance of endogenous fungi among the four groups of samples were as follows: Nectriella, Rhizoctonia, Mortierella, etc., had the highest relative abundances in AEZ-R; Filobasidium, unclassified_o__Pleosporales, Chaetosphaeronema, etc., had the highest relative abundances in AEZ-L; unclassified_o__Helotiales, Oliveonia, Exophiala, etc., had the highest relative abundances in AEY-R; unclassified_f__Mycosphaerellaceae, Sampaiozyma, Comoclathris, etc., had the highest relative abundances in AEY-L; Vishniacozyma, unclassified_f__Leptosphaeriaceae, unclassified_o__Filobasidiales, etc., had high relative abundances in leaf tissue; Dactylonectria, Mortierella, Veronaea, etc., had high relative abundances in root tissue. The differences in the community structure and abundance of endophytic bacteria in the four groups were as follows: Pedobacter had the highest relative abundance in AEZ-R; Sphingomonas, Rathayibacter, unclassified_f__Chitinophagaceae, etc., had the highest relative abundances in AEZ-L; Phyllobacterium, Mesorhizobium, Bosea, etc., had the highest relative abundances in AEY-R; Rhodococcus had the highest relative abundance in AEY-L. Pseudomonas and Phyllobacterium had high relative abundances in root tissue; and Methylobacterium-Methylorubrum, Rhodococcus, Rathayibacter, etc., had high relative abundances in leaf tissue. The relative abundances of norank_f__norank_o__Chloroplast and norank_f__Mitochondria were high in the four groups of samples.

#### 2.1.5. Analysis of Endophytic Biomarkers in the Root and Leaf Tissues of Cultivated and Wild-Type *A. euchroma*

**Figure 9:**
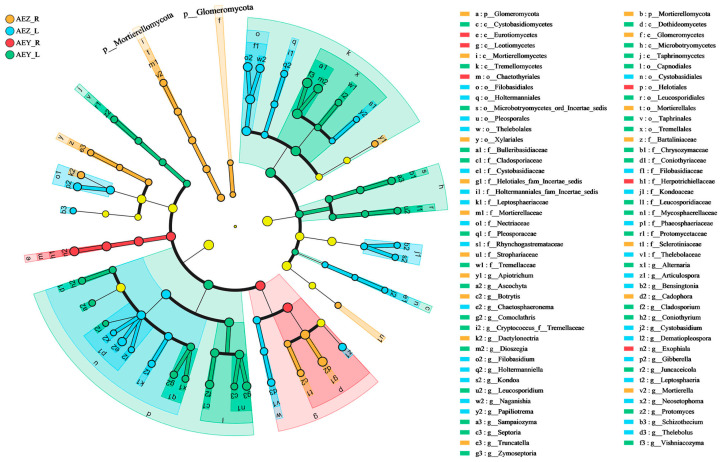
LEfSe analysis of *A. euchroma* endophytic fungal biomarkers.

**Figure 10:**
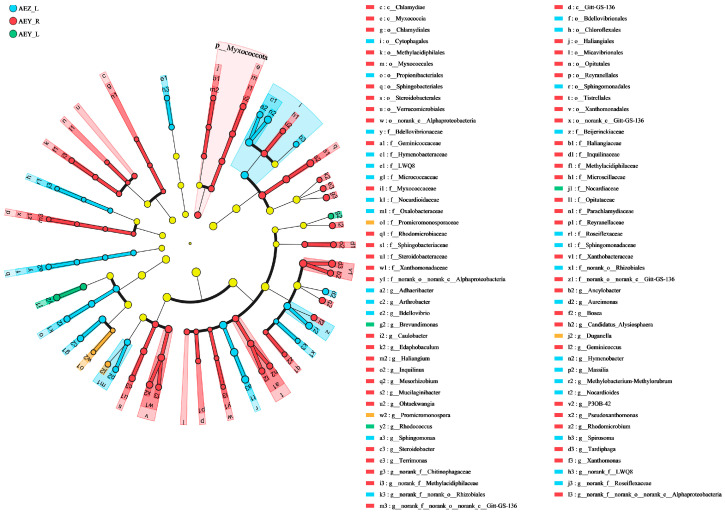
LEfSe analysis of *A. euchroma* endophytic bacterial biomarkers.

As endophyte indicators, endophytic biomarkers can accurately distinguish the types of endophytes in each group of samples, elucidate the patterns of community composition changes, and exert an impact on functions such as the metabolism of host plants. In this study, the LEfSe technique was utilized to explore endophytic biomarkers, aiming to identify indicator endophytes in cultivated and wild *A. euchroma* as well as in different tissues of *A. euchroma*. This was carried out to further explain the differences in the microbial community structure and ecological functions of the samples. In this study, the LEfSe technique [18] was employed to identify the differentially abundant microbial communities in the root and leaf tissues of cultivated and wild *A. euchroma*. Among the endophytes, an examination was carried out to find biomarkers with statistically significant differences at the phylum, class, order, family, and genus levels (Figure 9). At the phylum level, Mortierellomycota and Glomeromycota were significantly enriched in AEZ-R. At the genus level, Mortierella, Truncatella, Dactylonectria, etc., were significantly enriched in AEZ-R; Gibberella, Schizothecium, Neosetophoma, etc., were significantly enriched in AEZ-L; Exophiala was significantly enriched in AEY-R; and Protomyces, Coniothyrium, Ascochyta, etc., were significantly enriched in AEY-L. The LDA classification histogram (LDA > 2) is shown in Figure S1. The endophytic fungus f__Helotiales_fam_Incertae_sedis had the greatest influence on AEZ-R, o__Pleosporales had the greatest influence on AEZ-L, o__Helotiales had the greatest influence on AEY-R, and c__Tremellomycetes had the greatest influence on AEY-L. Among the endophytic bacteria (Figure 10), Myxococcota was significantly enriched in AEY-R at the phylum classification level. At the genus level, Promicromonospora and Duganella were enriched in AEZ-R; g__norank_f__LWQ8, g__norank_f__Roseiflex, Bdellovibrio, etc., were enriched in AEZ-L; g__norank_f__Methylacidiphilaceae, g__norank_f__norank_o__norank_c__Gift-Gs-136, Steroidobacter, etc., were enriched in AEY-R; and Rhodococcus and Brevaundimonas were enriched in AEY-L. An LDA histogram analysis (LDA > 2) showed that the endophytic bacteria belonging to g__Promicromonospora had the greatest influence on AEZ-R, o__Sphingomonadales had the greatest influence on AEZ-L, f__Xanthobacteraceae had the greatest influence on AEY-R, and f__Nocardiaceae had the greatest influence on AEY-L (Figure S2).

#### 2.1.6. Functional Prediction Analysis of *A. euchroma endophytes* with FUNGuild and PICRUSt2

**Figure 11:**
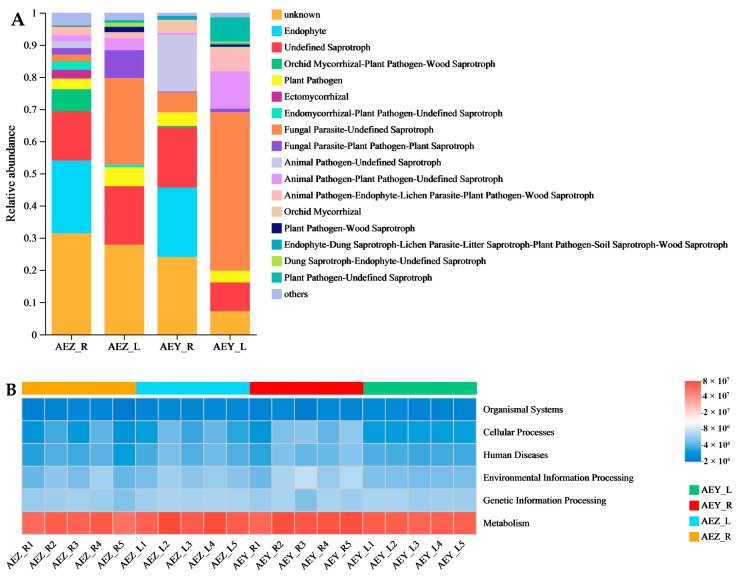

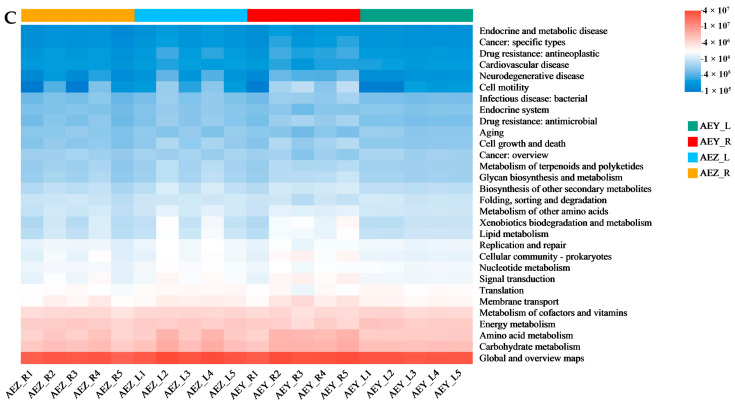
Functional prediction of endophytic fungi (**A**) with FUNGuild and endophytic bacteria with PICRUSt2 Level 1 (**B**) and Level 2 (**C**) in *A. euchroma* samples.

FUNGuild was used to predict the functions of the endophytic fungi from the root and leaf tissues of cultivated and wild *A. euchroma* (Figure 11). The results revealed that the endophytes could be divided into six model nutrient groups: pathotrophic, saprotrophic, symbiotic, pathosaprotrophic, saprobiotic, and pathosaprobiotic types. ASVs that did not match any taxa in the database were classified as others. AEZ-R was dominated by the saprotrophic and symbiotic types; AEZ-L was dominated by the saprotrophic and pathosaprotrophic types; AEY-R was dominated by the saprotrophic, symbiotic and pathosaprotrophic types; and AEY-L was dominated by the pathosaprotrophic type. In addition, a large proportion of bacteria with unknown functions were present in the four groups of samples, and their functions need to be further explored. The endophytic bacterial ASVs were compared against the KEGG, MetaCyc, and COG databases, and PICRUSt2 was used to predict the functions of the endophytic bacteria. As shown in Figure 11, the main functions of endophytic bacteria in the root and leaf tissues of *A. euchroma* were metabolism, genetic information processing, environmental information processing, human diseases, cellular processes, and organismal systems. Metabolism played a major role in the four groups of samples. At KEGG level 2, 10 metabolic pathways were identified: global and overview maps; carbohydrate metabolism; amino acid metabolism; energy metabolism; metabolism of cofactors and vitamins; membrane transport; translation; signal transduction; nucleotide metabolism; and the cellular community–prokaryotes. Among these pathways, the global and overview map pathways were the most prominent. However, most of these pathways are metabolic pathways.

### 2.2. Metabolomic Analysis of Different Tissues (Root and Leaf Tissues) in Cultivated and Wild A. euchroma

#### 2.2.1. Nontargeted Metabolomic Analysis of the Root and Leaf Tissues of Cultivated and Wild *A. euchroma*

**Figure 12:**
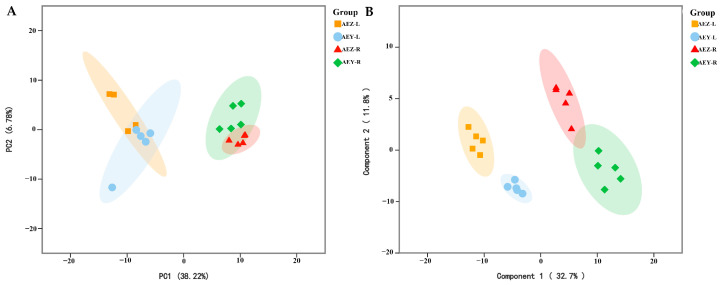
PCA (**A**) and OPLS-DA (**B**) of metabolites in *A. euchroma* samples. Each point in the diagram represents a sample, and samples from the same group are represented by the same color and shape.

The LC–MS/MS technique was used for qualitative analysis of the four groups of samples, and 248 metabolites were identified (Table S1). The metabolites identified included quinones, flavonoids, alkaloids, organic acids, sugars, amino acids, coumarins, sterols, terpenes, polyphenols, and fatty ketones and their derivatives. According to the statistical analyses, there were six naphthoquinone components among the top 20 metabolites in terms of their relative content in AEZ-R and AEY-R; the main component of the root tissue was naphthoquinone. Flavonoid components, such as rutin and quercetin, were among the top 20 metabolites in terms of relative content in AEZ-L and AEY-L; the main component of the leaf tissue was flavonoids. Principal component analysis (PCA) of all identified metabolites showed that differences in tissue type could account for 38.22% of the variation, while genotype-specific differences could account for 6.78% of the variation. The 20 samples were clearly divided into four groups, indicating significant metabolic differences. Further observation revealed that in the PCA plot (Figure 12A), AEZ-L and AEY-L were close, and AEZ-R and AEY-R were close, whereas the distance between roots and leaves was large, indicating significant metabolic differences between the different tissues. In addition, the distance between cultivated and wild *A. euchroma* was small, but they were separate, indicating that there were also metabolic differences between cultivated and wild *A. euchroma*. Orthogonal partial least squares discriminant analysis (OPLS-DA) was used to distinguish the four groups according to their metabolic characteristics, further revealing the metabolic differences among the four groups of samples (Figure 12B). In addition, the relative contents of 7-hydroxycoumarin, *α*-linolenic acid, salvianolic acid B, raffinose, trehalose, quercetin, rutin, naringenin, and palmitic acid were relatively high in AEZ-L and AEY-L. The relative contents of *β*-dimethylacrylylakanin, akanin, propionylshikonin, medicarpin, raffinose, deoxyshikonin, methyl heptadecanoate, acetylshikonin, isovalerylshikonin, and palmitic acid were relatively high in AEZ-R and AEY-R. Differentially abundant metabolites were screened according to two criteria (VIP > 1, *p* < 0.05). The analysis revealed that 104 (Table S2) metabolites were differentially abundant between AEZ-R and AEZ-L (74 upregulated, 30 downregulated). Eighty-two (Table S3) differentially abundant metabolites were screened between AEY-R and AEY-L (55 upregulated, 27 downregulated), and 6 (Table S4) differentially abundant metabolites were screened between AEZ-R and AEY-R (4 upregulated, 2 downregulated). Nine differentially abundant metabolites (Table S5) were screened between AEZ-L and AEY-L (up = 9).

#### 2.2.2. Determination of the Contents of Eight Naphthoquinones in Cultured and Wild *A. euchroma* Root Tissues by HPLC

**Figure 13:**
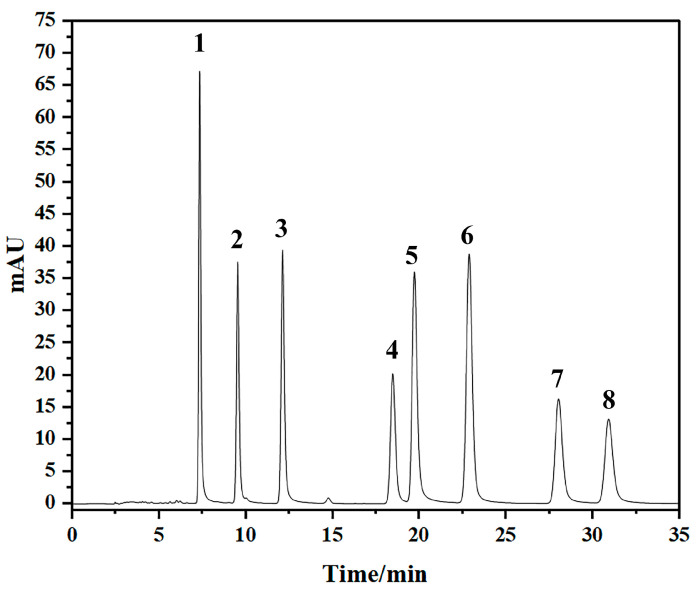
HPLC chromatograms of the eight naphthoquinone components of *A. euchroma*. 1: L-shikonin; 2: *β*-hydroxy-isovalerylakanin; 3: acetylshikonin; 4: *β*-acetoxy-isovalerylakanin; 5: deoxyshikonin; 6: isobutyrylshikonin; 7: *β,β’*-methylacrylylakanin; 8: isovaleryshikonin.

In accordance with the methods and conditions described in the “Chinese Pharmacopoeia, 2020”, the contents of eight naphthoquinones in AEZ-R and AEY-R were determined via HPLC. The HPLC standards for the eight secondary metabolites of *A. euchroma* are shown in Figure 13, indicating that the eight secondary metabolites of *A. euchroma* could be effectively detected under these HPLC conditions. The HPLC chromatograms of the samples can be seen in Figure S3. As shown in Table S6, the *β,β’*-dimethylacrylylakanin contents of cultivated and wild *A. euchroma* were compared; the *β,β’*-dimethylacrylylakanin content of wild *A. euchroma* was greater than that of cultivated *A. euchroma*, indicating that there was a difference in quality between cultivated and wild *A. euchroma*.

### 2.3. Correlation Analysis of Microbiomes and Metabolomes

**Figure 14:**
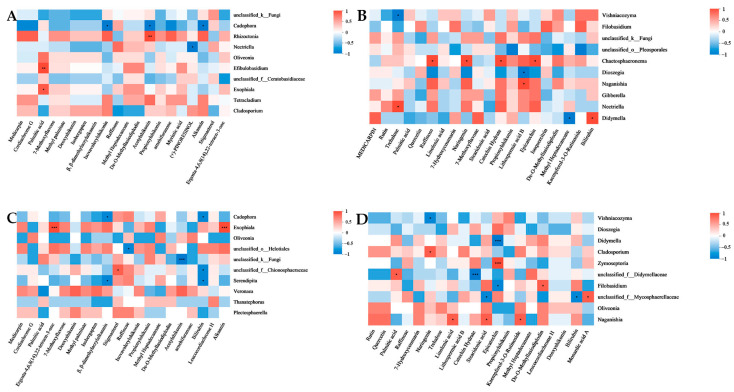
Heatmaps of the correlations between endophytic fungi and metabolites in *A. euchroma* samples. (**A**) AEZ-R; (**B**) AEZ-L; (**C**) AEY-R; (**D**) AEY-L (*p* values less than 0.05 are indicated by *; * 0.01 < *p* ≤ 0.05; ** 0.001 < *p* ≤ 0.01; *** *p* ≤ 0.001). Red represents positive correlations, and blue represents negative correlations.

**Figure 15:**
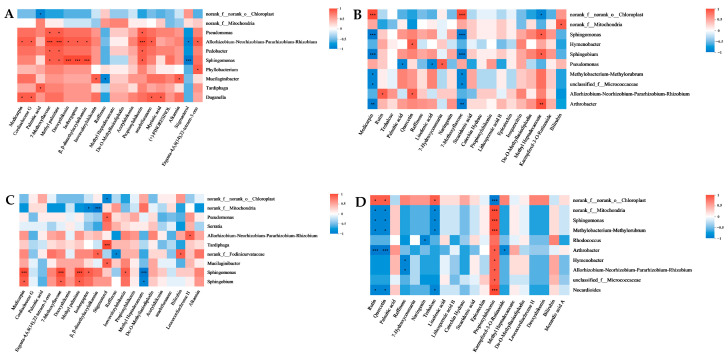
Heatmaps of the correlations between endophytic bacteria and metabolites in *A. euchroma* samples. (**A**) AEZ-R; (**B**) AEZ-L; (**C**) AEY-R; (**D**) AEY-L (*p* values less than 0.05 are indicated by *; * 0.01 < *p* ≤ 0.05; ** 0.001 < *p* ≤ 0.01; *** *p* ≤ 0.001).

A Spearman correlation heatmap was employed to analyze the correlations between the relative abundances of the top 10 ASVs of endophytic fungi and endophytic bacteria at the genus level and the relative contents of the top 20 metabolites in the root and leaf tissues of cultivated and wild *A. euchroma*. The results revealed that several of the dominant endophytic fungi among the endogenic fungi were significantly correlated with the dominant metabolites (Figure 14). Among them, Cadophora in AEZ-R was negatively correlated with several dominant metabolites. Chaetosphaerema in AEZ-L was positively correlated with many dominant metabolites. There was a significant positive correlation between Exophiala and several dominant metabolites in AEY-R. Naganishia in AEY-L was positively correlated with several dominant metabolites. In addition, there were significantly more dominant endophytic fungal species in the wild plants than in the cultivated plants, and there were more dominant endophytic fungal species in the leaf tissue than in the root tissue. A variety of dominant endophytic bacteria were significantly correlated with the dominant metabolites of the endophytic bacteria (Figure 15). Allorhizobium–Neorhizobium–Pararhizobium–Rhizobium and Sphingomonas in AEZ-R were significantly positively correlated with many dominant metabolites. Arthrobacter in AEZ-L was positively and negatively correlated with many dominant metabolites.

There were significant positive correlations between Sphingobium and several dominant metabolites in AEY-R. Arthrobacter in AEY-L was significantly positively and negatively correlated with many dominant metabolites. In addition, all 10 dominant endophytic bacteria in AEY-L, except Rhodococcus, were significantly positively correlated with propionylshikonin.

**Figure 16:**
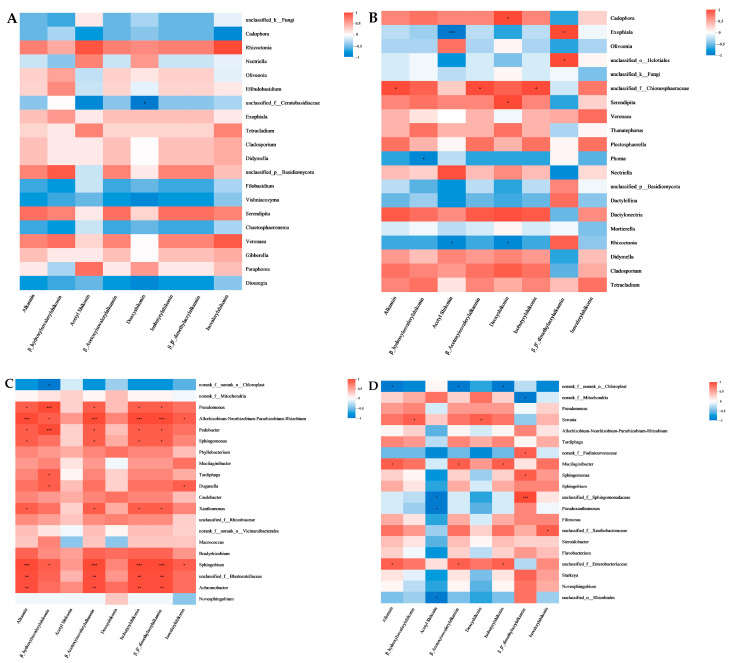
Heatmaps of the correlations between the contents of endophytic fungi in (**A**) AEZ-R and (**B**) AEY-R, endophytic bacteria in (**C**) AEZ-R and (**D**) AEY-R and eight naphthoquinones in *A. euchroma* samples. (*p* values less than 0.05 are represented by *; * 0.01 < *p* ≤ 0.05, ** 0.001 < *p* ≤ 0.01, and *** *p* ≤ 0.001.).

Spearman correlation heatmaps were used to analyze the correlations between the relative abundances of the top 20 ASVs of endophytic fungi and endophytic bacteria at the genus level and the chemical components of eight naphthoquinones in AEZ-R and AEY-R. Among the endophytic fungi, only unclassified_f__Ceratobasidiaceae in AEZ-R (Figure 16A) presented a significant negative correlation with deoxyshikonin. In AEY-R (Figure 16B), unclassified_f __Ceratobasidiaceae and alkannin, *β*_acetoxyisovalerylalkannin, and isobutyrylshikonin were significantly positively correlated. Among the endophytic bacteria in AEZ-R (Figure 16C), Pseudomonas, Allorhizobium–Neorhizobium–Pararhizobium–Rhizobium, Pedobacter, Sphingomonas, Xanthomonas, Sphingobium, unclassified_f__Blastocatellaceae, and Achromobacter were significantly positively correlated with many naphthoquinones. In AEY-R (Figure 16D), norank_f__norank_o__Chloroplast presented significant negative correlations with many naphthoquinones. Mucilaginibacter and unclassified_f__Enterobacteriaceae were positively correlated with many naphthoquinones. In addition, the figure intuitively shows that the flora significantly related to the naphthoquinone components included more endophytic bacteria than endophytic fungi.

## 3. Discussion

The distribution of endophytic bacteria in medicinal plants is characterized by universality and diversity. Endophytes have been found in all medicinal plants studied thus far, and the diversity of endophytes in plants is closely related to the species, whether the plants are cultivated or wild, their growth environment, their growth stage, the nutrient supply, and the genotype of the plants. The diversities of endophytes in the tissues of the same plant growing in different regions and different environments, and even in different parts of tissues of the same plant in the same region, are significant [19]. In this study, endophytes were abundant in cultivated and wild *A. euchroma* and in different tissues. The α-diversity index analysis revealed that the community structure and composition of endophytes showed significant tissue specificity, especially the diversity of endophytic fungi, which was greater in cultivated *A. euchroma* than in wild *A. euchroma* and was greater in leaf tissue than in root tissue. Moreover, PCoA and NMSD analyses revealed a clear boundary between leaf tissue and root tissue, which once again verified the tissue specificity of *A. euchroma* endophytes. There were also differences in endophyte diversity between cultivated and wild *A. euchroma*, but these differences were not significant, possibly because the wild and cultivated composts came from only one population each and had similar habitats; thus, increasing the completeness of the data will be necessary in the future. This study explored the differences between different growth patterns and tissue types of *A. euchroma* from the perspective of the microbiome to provide a basis for clarifying the mechanism by which the endophytic flora affects the quality of Arnebia Radix.

Studies have shown that endophytic fungi are important natural decomposers that effectively decompose cellulose and lignin [20]. Among them, Ascomycetes and Basidiomycetes are the dominant fungi in most soils and plants. They can also participate in the carbon cycle by degrading organic matter [21]. In this study, the dominant endophytic fungi in the root and leaf tissues of cultivated and wild *A. euchroma* were Ascomycota and Basidiomycota, which may have helped *A. euchroma* adapt to the soil environment and participate in nutrient cycling. The dominant endophytic bacteria are Proteobacteria and Cyanobacteria, which play important roles in nature. Most of these bacteria are related to the nitrogen and sulfur cycles [22], play important roles in the conversion and utilization of carbon and nitrogen, and have functions such as fixing heavy metals, dissolving phosphorus, and degrading hydrocarbons. However, nitrogen, phosphorus, and other elements are essential nutrients for plant growth. Cyanobacteria have a strong metabolic capacity. Through photosynthesis, nitrogen fixation, and other processes, they produce a rich variety of metabolites, which can improve soil fertility and promote plant growth. Cyanobacteria play an important role in helping plants better adapt to stress, improving their physiological and biochemical functions, and optimizing their growth characteristics, yield components, and photosynthetic efficiency [23]. As the dominant endophytic bacteria of *A. euchroma*, cyanobacteria may participate in or influence the synthesis and accumulation of metabolites and play a crucial role in the production of some beneficial metabolites. Proteobacteria and cyanobacteria exist in *A. euchroma* tissues in large quantities, providing necessary nutrients for the growth of *A. euchroma* and its resistance to abiotic stress and invasion by plant pathogens. The results of this study indicate that there is a selective specificity between endophytes and host plants, which is consistent with the research findings of Rai et al. [24,25,26]. *A. euchroma* recruits beneficial flora and provides a suitable environment for the growth and reproduction of the flora. Similarly, the flora provide nutrients and help *A. euchroma* resist stress, playing an important role in plant growth and health; this relationship may be the result of natural selection and coevolution between microorganisms and host plants. This study provides a theoretical basis for further research on the mechanisms underlying the interactions between microbes and host plants and for screening *A. euchroma* growth-promoting endophytes.

LEfSe analysis can identify species at different classification levels and screen for significant differences, for example, through the examination of biomarkers with significant differences. In this study, the most significant biomarkers in AEY-L were the endophytic fungus Tremellomycetes and the endophytic bacterium f__Nocardiaceae. The most significant biomarkers in AEY-R were the endophytic fungus o__Helotiales and the endophytic bacterium f__Xanthobacteraceae. The most significant biomarkers in AEZ-L were the endophytic fungus __Pleosporales and the endophytic bacterium f__Sphingomonadaceae. The most significant biomarkers in AEZ-R were the endophytic fungus Spendomyces dorsalis and the endophytic bacterium Mucilaginibacter defluvii. Among these fungi, the endophytic fungus c__Tremellomycetes belongs to Basidiomycota, and o__Helotiales and g__Cadophora belong to Ascomycota. These two dominant phyla may play important roles as biomarkers in *A. euchroma* tissue. The endophytic bacterium f__Xanthobacteraceae belongs to the order Rhizobia, and some bacteria in the order Rhizobia play roles in nitrogen fixation. Some bacteria in the f__Sphingomonadaceae family affect the physiological metabolism of plants and increase the resistance of plants to disease and insects [27]. Studies have shown that f__Sphingomonadaceae can produce heat shock proteins and improve the cold, heat, and drought tolerance of plants [28]. These biomarkers may be key to the healthy growth and resistance of *A. euchroma* under stress and foreign invasion. Future studies will focus on these flora, screen and optimize the core functional microbial mix required for the cultivation of *A. euchroma*, and address problems such as slow growth and readily occurring root rot during the cultivation of *A. euchroma*.

PICRUSt2 is a reliable tool for predicting the metabolic functions of bacterial communities [29,30], and the analysis revealed a total of six functional pathways, including metabolism, genetic information processing, environmental information processing, human diseases, cellular processes, and organismal systems. In the field of metabolism, pathways such as amino acid metabolism, cofactor and vitamin metabolism, nucleotide metabolism, carbohydrate metabolism, and energy metabolism were identified. In addition, pathways related to cellular processes (such as the prokaryotic cell community), environmental information processing pathways (such as membrane transport and signal transduction), and genetic information processing pathways (such as translation) were also identified. Notably, the three pathways with the highest relative abundance in all the samples except for the global and overview map pathways were carbohydrate metabolism, amino acid metabolism, and energy metabolism. These results are consistent with the prediction of the functions of the endophytic bacteria of *Panax quinquefolius* L. [13]. FUNGuild has been used to identify and compare the specific functions of fungi in recent years. In this study, FUNGuild software was used to predict the functions of the endophytic fungi of *A. euchroma*, and six nutritional model groups were identified: pathophysiotrophic, saprotrophic, symbiotic, pathophysiotrophic, saprobiotrophic, and pathophysiotrophic. PICRUSt2 can predict various functions of endophytic bacteria, but further research is needed to verify the underlying mechanisms involved. FUNGuild predicts the nutrient types of endophytic fungi, but it is not comprehensive enough and has limitations, and a joint analysis of the rhizosphere and soil microorganisms of *A. euchroma* is needed to explore their specific functions.

*A. euchroma* is rich in chemical components with significant biological activities, such as amino acids, phenolic compounds, and their derivatives [31], which have anti-inflammatory, antibacterial, antitumor, wound-healing, and other pharmacological effects. To date, many studies on *A. euchroma* metabolites have focused mostly on naphthoquinones in root tissues. In this study, nontargeted metabolomics of the root and leaf tissues of cultivated and wild *A. euchroma* were carried out, and 248 metabolites were identified in positive ion mode. The root tissue of *A. euchroma* mainly contains naphthoquinone components, whereas the leaf tissue mainly contains flavonoids. PCoA and OPLS-DA revealed differences between cultivated and wild *A. euchroma* and between root and leaf tissues. Moreover, according to the analysis (VIP > 1, *p* < 0.05), cultivated and wild-type *A. euchroma* differed as follows: nine differentially abundant metabolites were screened between AEZ-L and AEY-L, and six differentially abundant metabolites were screened between AEZ-R and AEY-R. However, the differences between the different tissues were more significant: 104 differentially abundant metabolites were identified between AEZ-R and AEZ-L, and 82 differentially abundant metabolites were identified between AEY-R and AEY-L. Thus, the metabolites also exhibited tissue specificity in *A. euchroma*. The results of this study are consistent with those of previously reported studies; for example, the metabolites in different tissues of *Panax notoginseng* significantly differ [32]. Moreover, the relative content of flavonoids in the leaf tissue of *A. euchroma* was relatively high, which is consistent with previous reports [33]. Naphthoquinones were the main components in root tissues. HPLC was used to measure the contents of eight naphthoquinones in cultivated and wild *A. euchroma* root tissues. The results revealed that the content of *β,β’*-dimethylacrylylakanin in wild *A. euchroma* was greater than that in cultivated *A. euchroma*, indicating a difference in quality between cultivated *A. euchroma* and wild *A. euchroma*. In summary, this study was the first to conduct nontargeted metabolomics on root and leaf tissues of cultivated and wild *A. euchroma* and to analyze the differences in all metabolites across different growth environments and different tissue parts to improve the comprehensive utilization value of *A. euchroma* resources.

Endophytes can regulate the accumulation of secondary metabolites in medicinal plants and can be used not only as reservoirs of new bioactive secondary metabolites but also as potential substitutes for secondary metabolites in medicinal plants. In this study, a combination of metabolomics and microbiological analysis was employed and revealed that the dominant flora in the four groups of samples were significantly correlated with a variety of metabolites. These results once again indicate that endophytes play an important role in the accumulation of secondary metabolites in medicinal plants and that endophytes may have different effects on the accumulation of secondary metabolites in different plants. For example, Pseudomonas in AEZ-L was significantly positively correlated with 7-hydroxycoumarin but significantly negatively correlated with linolenic acid. The results revealed that there were more endophytic fungi in wild *A. euchroma* than in cultivated *A. euchroma* and more endophytic fungi in leaf tissues than in root tissues, indicating that more endophytic fungi in wild *A. euchroma,* and more endophytic fungi in leaf tissues, participated in the accumulation of metabolites. The relative abundances of all the top 10 endophytic bacteria in AEY-L, except Rhodococcus, were significantly positively correlated with propionylshikonin, indicating that the endophytic bacteria in AEY-L may have had a relatively strong influence on the propionylshikonin level. These results provide a theoretical basis for the participation of endophytes in the accumulation of metabolites in *A. euchroma* and for the search for alternative medicinal plant resources. In this study, the relationships between the diversity of endophytes in cultivated and wild *A. euchroma* root tissues and the contents of eight naphthoquinones were further investigated through heatmap analysis. The analysis revealed that eight naphthoquinones in cultivated and wild root tissues were significantly correlated with a variety of dominant endophytes, which was similar to the results from research on *Rheum palmatum* and *Cynomorium songaricum* Rupr [14,34], and there were also significant correlations between the corresponding metabolites and endophytic fungi. Moreover, there were significant positive correlations between the endophytic fungi Exophiala and unclassified_o__Helotiales in AEY-R and the indicator component *β,β’*-dimethylacrylakanin in Arnebia Radix. A variety of endophytic bacteria also showed significant positive correlations with the indicator component *β,β’*-dimethylacrylakanin in Arnebia Radix, and Pseudomonas, Allorhizobium–Neorhizobium–Pararhizobium–Rhizobium, Pedobacter, Sphingomonas, Xanthomonas, Sphingobium, unclassified_f__Blastocatellaceae, Achromobacter, and the contents of four naphthoquinone components in Arnebia Radix showed significant positive correlations. These endophytic bacteria may be involved in the accumulation of naphthoquinone components in Arnebia Radix. Naphthoquinones are enriched in the cork of *A. euchroma*, and corking is the protective mechanism by which *A. euchroma* adapts to the environment. Thus, we concluded that these endophytes, which are significantly related to the naphthoquinone components, may affect the synthesis of these components, participate in the process of *A. euchroma* corking, and thus interfere with the growth and development of *A. euchroma*, which may be key to improving the quality of *A. euchroma*. In addition, unclassified_f__Ceratobasidiaceae, among the dominant endophytic fungi, was negatively correlated with many naphthoquinones in cultivated *A. euchroma* but positively correlated with many naphthoquinones in wild *A. euchroma*. These results indicate that unclassified_f__Ceratobasidiaceae might be responsible for the difference in quality between wild and cultivated *A. euchroma*. Moreover, there were more endophytic bacteria than endophytic fungi among the flora significantly related to naphthoquinones, which may indicate that more endophytic bacteria were involved in the accumulation of naphthoquinones in the root tissues of *A. euchroma*. This study provides a foundation for further research on the mechanism of *A. euchroma* corking and addresses the problems associated with the low degree of corking and the poor quality of *A. euchroma*. In future research, we will focus on related endophytes and work on isolating endophytes from *A. euchroma*, followed by reinoculating them into *A. euchroma* to verify their effects on the accumulation of metabolites in *A. euchroma* and the underlying mechanisms; these studies will help improve the quality of cultivated *A. euchroma*.

## 4. Materials and Methods

### 4.1. Experimental Materials

**Figure 17:**
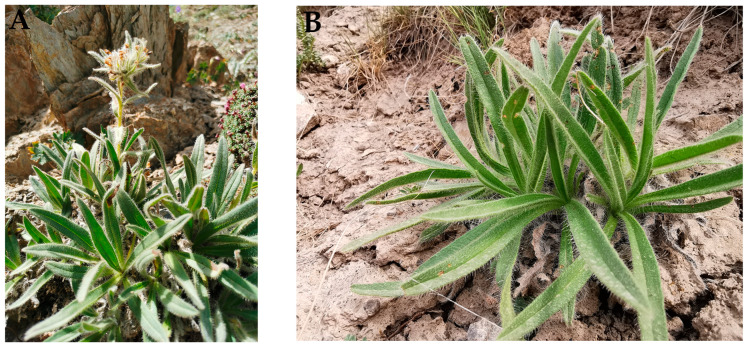

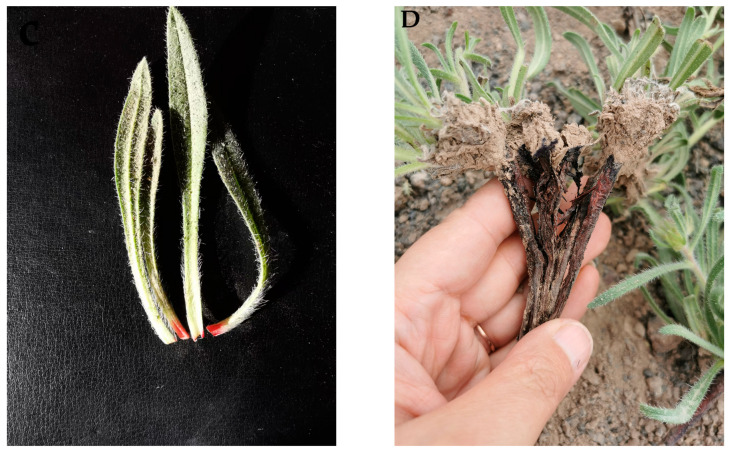
Pictures of *A. euchroma* plants (**A**,**B**). Leaf (**C**) and root (**D**) tissues of *A. euchroma* plants.

Fresh, healthy cultivated and wild *A. euchroma* samples were obtained from Hejing County, Bayingolin Mongolian Autonomous Prefecture, Xinjiang Uyghur Autonomous Region (Figure 17). The cultivated *A. euchroma* used in this study was collected for scientific research with the permission of the Hejing Ru Le Eco-Agricultural Cooperative (Urumqi, China), and the wild *A. euchroma* was collected with the permission of the Hejing County Forestry and Grassland Bureau. The voucher specimens were stored in the Traditional Chinese Medicine Herbarium of Xinjiang Medical University (voucher numbers: XJMU20230910001, XJMU20230910002) and were identified by Professor Haiyan Xu. The samples were divided into 4 groups and photographed: AEZ-R, AEZ-L, AEY-R, and AEY-L. Five biological replicates were examined in each group. The surface dirt and adhered materials were removed though successive rinsing with distilled water, immersion in 75% ethanol for 5 min and 1% NaClO for 3 min, and washing with sterile water 3 times. Finally, sterile water was inoculated on nutrient agar (NA) medium and potato dextrose agar (PDA) medium, followed by cultivation at 28 °C for 10 d and 37 °C for 5 d; sterile plates were generated to achieve surface disinfection [35]. *A. euchroma* samples were dried on sterile filter paper and placed in Ep tubes at −80 °C for subsequent high-throughput sequencing. The samples used for the metabolomics studies were dried, crushed, passed through sieve number six (100 eyes), and set aside.

### 4.2. DNA Extraction, PCR Amplification and 16S and ITS2 rRNA Sequencing Analysis of A. euchroma Endophytes

*A. euchroma* DNA extraction was performed using a DNA kit (Fast DNA^®^ SPIN Kit, MPBIO, Santa Ana, CA, USA). DNA was amplified through PCR on an ABI GeneAmp^®^ Model 9700. The ITS1 region of rDNA was amplified using the fungal primers ITS1F (5′-CTTGGTCATTTAGAGGAAGTAA-3′) and ITS2R (5′-GCTGCGTTCTTCATCGATG-C-3′) [36]. The reaction system volume was 20 µL and contained 10× Buffer (2 µL), 2.5 mM dNTPs (2 µL), 5 µmol/L forward primer and reverse primer (0.8 µL each), TaKaRa rTaq DNA polymerase (0.2 µL), BSA (0.2 µL), the DNA template (10 ng), and ddH_2_O up to 20 µL. The PCR procedure was as follows: initial denaturation at 95 °C for 3 min; 35 cycles of denaturation at 95 °C for 30 s, annealing at 55 °C for 30 s, and extension at 72 °C for 45 s; extension at 72 °C for 10 min; and storage at 10 °C. The PCR products were detected using 2% agarose gel electrophoresis. The bacterial primers 338F (5′-ACTCCTACGGGGAGGCAGCA-3′) and 806R (5′-GGACTACHVGGGTWTCTAAT-3′) were used to amplify the bacterial 16S gene (V3-V4 region). The reaction system volume was 20 µL, and contained 5× Buffer (4 µL), 2.5 mM dNTPs (2 µL), 5 µM each primer (0.8 µL each), FastPfu Polymerase, the DNA template (10 ng), and dH_2_O (10 µL). The PCR procedure was as follows: initial denaturation at 95 °C for 3 min; 35 cycles of denaturation at 95 °C for 30 s, annealing at 52 °C for 30 s, and extension at 72 °C for 45 s; a final extension at 72 °C for 5 min; and preservation at 10 °C [37]. The PCR products were also tested using 2% agarose gel electrophoresis. PCR amplification and Illumina MiSeq sequencing were performed by Shanghai Majorbio Co., Ltd., Shanghai, China (project number MJ20230614095). The successful PCR products from all the samples were pooled and purified using an EasyPure^TM^ PCR Cleanup/Gel Extraction Kit (Axygen Biosciences, Union City, CA, USA) according to the manufacturer’s instructions. All the samples were amplified and pooled three times before sequencing. The purified PCR products were sequenced on the Illumina NovaSeq platform [38]. The original data obtained were subjected to DADA2 (https://qiime2.org; accessed on 16 November 2023) to obtain single-base precision amplicon sequence variants (ASVs) through sequence correction. Mothur (version 1.30.2) software was used to calculate the alpha diversity index of each sample. The total number of endophytes in each group was calculated according to the relative abundance of endophytes, and a Venn diagram was constructed. The Kruskal–Wallis rank sum test was used to test the alpha diversity indices of the four groups of samples. The Bray–Curtis weighting algorithm was used to analyze the normalized ASV table, and QIIME2 was used to evaluate *β* diversity [39]. The nonmetric multidimensional scale sorting (NMDS) analysis method was used to find the classification differences among samples based on nonweighted distance measurements. PCoA diagrams were constructed on the Majorbio i-Sanger Cloud platform in R software (version 3.3.1). Using R software (3.3.1) on the Majorbio i-Sanger Cloud platform, the composition of endophytic bacteria at the phylum and genus levels was visually analyzed. The effects were examined by linear discriminant analysis (LEfSe, https://report.majorbio.com/asv/lefse/task_id/703o_fvgckpdlnq29hl66ob2m4t.html; accessed on 9 September 2024). In LEfSe, taxonomic groups with LDAs greater than 2 are identified as biomarkers, thus revealing the impact of the signature species identified between different groups on the differential effect [40]. The FUNGuild (http://www.funguild.org/; accessed on 9 September 2024) and PICRUSt2 (http://huttenhower.sph.harvard.edu/galaxy; accessed on 9 September 2024) databases were used to predict the ecological functions of endophytic fungi and bacteria, respectively [41]. Analysis of variance (ANOVA) and the Kruskal–Wallis rank sum test were used for all the statistical tests, and the level of significance at *p* < 0.01 was the least significant difference.

### 4.3. Metabolomics Analysis

#### 4.3.1. Nontargeted Metabolomics Analysis of the Root and Leaf Tissues of Cultivated and Wild *A. euchroma*

A total of 0.5 g of each of the AEZ-R, AEZ-L, AEY-R, and AEY-L samples was precisely weighed and placed in a 50 mL centrifuge tube. Next, 10 mL of methanol was added, and the mixture was subjected to ultrasonic extraction at 200 Hz for 30 min. The mixture was subsequently centrifuged at 1000 rpm for 10 min, after which the supernatant was collected. The supernatant was evaporated to dryness in a water bath at 60 °C. The residue was dissolved in 5 mL of chromatographic methanol to obtain the sample stock solution. The stock solution was diluted and filtered through a 0.22 μm microporous membrane to obtain the test solution. The mobile phase consisting of 0.1% formic acid aqueous solution (A) and acetonitrile (B) was injected into the column (ACQUITYUPLC BEH C18, Waters, Milford, MA, USA). The linear gradients were as follows: 0–1 min, 90% A; 1–35 min, 5% A; 35–36 min, 5% A; 36–38 min, 90% A; and 38–40 min, 90% A. The column temperature was 40 °C, the flow rate was 0.3 mL/min, and the injection volume was 5 µL. The Q-TOF (Waters) system uses an ESI ion source to collect data in positive ion mode. The data collection range was 100–1500 *m*/*z*. The ion source parameters were as follows: capillary voltage, 3.0 kV (positive ion mode); sample cone voltage, 55 V. By extracting the ion flow, qualitative analysis of the precise mass number was carried out, the molecular weight error was less than 10 ppm, the possible molecular formula was screened, and the compound of the composition was further determined based on the fragment information via tandem mass spectrometry in positive ion mode, combined with the literature reports.

Principal component analysis (PCA) was performed on the identified metabolites, and orthogonal partial least squares discriminant analysis (OPLS-DA) was used to distinguish the groups according to their metabolic characteristics. After data normalization, an OPLS-DA model was constructed. Differentially abundant metabolites were screened according to two criteria: VIP > 1 and *p* < 0.05.

#### 4.3.2. Determination of the Levels of 8 Naphthoquinones by HPLC

With reference to the 2020 edition of the Chinese Pharmacopoeia, the contents of 8 naphthoquinones (L-shikonin, *β,β’*-dimethylacrylylakanin, deoxyshikonin, acetylshikonin, *β*-acetoxyisovalerylakanin, *β*-hydroxyisovalerylakanin, isobutylshikonin, and isovalerylshikonin) were determined by high-performance liquid chromatography. The method was as follows: samples of the AEZ-R and AEY-R powders (0.5 g) were precisely weighed and placed in conical bottles, and 25 mL of petroleum ether was precisely measured and added. After the medicinal materials were thoroughly filtered, ultrasonication was performed for 30 min, and the resulting weight loss was compensated for by cooling, after which the mixture was filtered. Ten milliliters of the remaining filtrate was removed, placed in an evaporating dish, dried in a water bath at 55 °C, dissolved with chromatographic acetonitrile, and finally transferred to a 10 mL volumetric bottle containing chromatographic acetonitrile to obtain the test solution. An Agilent 1260 instrument and an Agilent-C18 column (4.6 × 250 mm, 5 µm) were used for high-performance liquid chromatography. The mobile phase was an acetonitrile-0.05% formic acid–water (70:30) mixture, the flow rate was 1 mL/min, the column temperature was 30 °C, the detection wavelength was 275 nm, and the sample volume was 10 µL. The contents of the 8 naphthoquinones in all the samples were determined using the standard curve obtained from previous data of the research group [42].

### 4.4. Correlation Analysis

Pearson correlation analysis in SPSS 23.0 software was used to establish the correlation between the relative abundance of endophytes of dominant genera and the relative contents of metabolites in cultivated and wild *A. euchroma* and in root and leaf tissues at the genus level. The correlations between the dominant endophytes in cultivated and wild A. euchroma root tissues and the contents of 8 naphthoquinones in the root tissues were also analyzed. A correlation heatmap was drawn to directly show the correlations between the dominant flora and metabolites in the samples.

## 5. Conclusions

In summary, the endophytic community diversity of cultivated *A. euchroma* was different to that of wild *A. euchroma*, and the endophytes presented tissue specificity in *A. euchroma*. Proteophyta, Cyanophyta, Ascomycetes, and Basidiomycetes were the dominant endophytes in the *A. euchroma* samples. The *A. euchroma* metabolites exhibited tissue specificity, and there was no significant difference in metabolites between cultivated and wild *A. euchroma*. Various metabolites of *A. euchroma* were positively and negatively correlated with dominant endophytes, and the main active substances of *A. euchroma* were also significantly correlated with endophytes. These results provide an important basis for exploring microbial–metabolite–host–plant interactions and are highly important for mining beneficial microbial resources from *A. euchroma*.

## Figures and Tables

**Table 1:** Sample valid sequence statistics.

Strains/Samples	AEZ-R	AEZ-L	AEY-R	AEY-L	Total
Endophytic fungi	262,092	432,383	253,483	363,260	1,311,218
Endophytic bacteria	215,224	254,729	237,869	229,799	937,621

**Table 2:** Alpha diversity indices of the samples.

Sample	Endophytic Fungi				Endophytic Bacteria			
	ASV	Shannon	Chao	Goods Coverage	ASV	Shannon	Chao	Goods Coverage
AEZ-R	542	2.182	165.949	0.999	749	1.369	184.936	0.999
AEZ-L	584	3.299	226.775	0.999	2343	2.575	628.528	0.998
AEY-R	324	1.986	86.019	0.999	2143	3.170	511.697	0.998
AEY-L	516	2.991	178.441	0.999	1193	1.207	314.954	0.998

Note: All the data in the table are the average values of five sets of parallel experiments.

## Data Availability

The sequencing data generated in the study are deposited in the NCBI SRA database under BioProject Nos. PRJNA1178297 and PRJNA1178299.

## Supplementary Materials

The following supporting information can be downloaded at https://www.mdpi.com/article/10.3390/molecules30030734/s1, Figure S1: Bar plots of LDA scores of endophytic fungi in different tissues (roots and leaves) of cultivated and wild A. euchroma, showing only taxa that met the LDA significance threshold > 2. Figure S2: Bar plots of LDA scores of endophytic bacteria in different tissues (roots and leaves) of cultivated and wild A. euchroma, showing only taxa that met the LDA significance threshold > 2. Table S1: Total metabolites of positive ion patterns identified from different tissue parts of cultivated and wild A. euchroma. Table S2: Different metabolites between AEZ-R and AEZ-L; Table S3: Different metabolites between AEY-R and AEY-L; Table S4: Different metabolites between AEZ-R and AEY-R; Table S5: Different metabolites between AEZ-L and AEY-L; Table S6: The eight naphthoquinone components contained in AEZ-R and AEY-R (mg/g).

## Abbreviations

The following abbreviations are used in this manuscript:

*A. euchroma*	*Arnebia euchroma* (Royle) Johnst.
ASV	Amplicon sequence variant
UPLC–MS	Ultra-high-performance liquid chromatography–mass spectrometry
KEGG	Kyoto Encyclopedia of Genes and Genomes
