# Supplementary figures and images for: Study of the Correlation Between Endophyte Abundances and Metabolite Levels in Different Parts of the Tissue of Cultivated and Wild Arnebia euchroma (Royle) Johnst. Based on Microbiome Analysis and Metabolomics
Source: Molecules. 2025 Feb 6;30(3):734. doi: 10.3390/molecules30030734 (PMC11820562; doi:10.3390/molecules30030734)

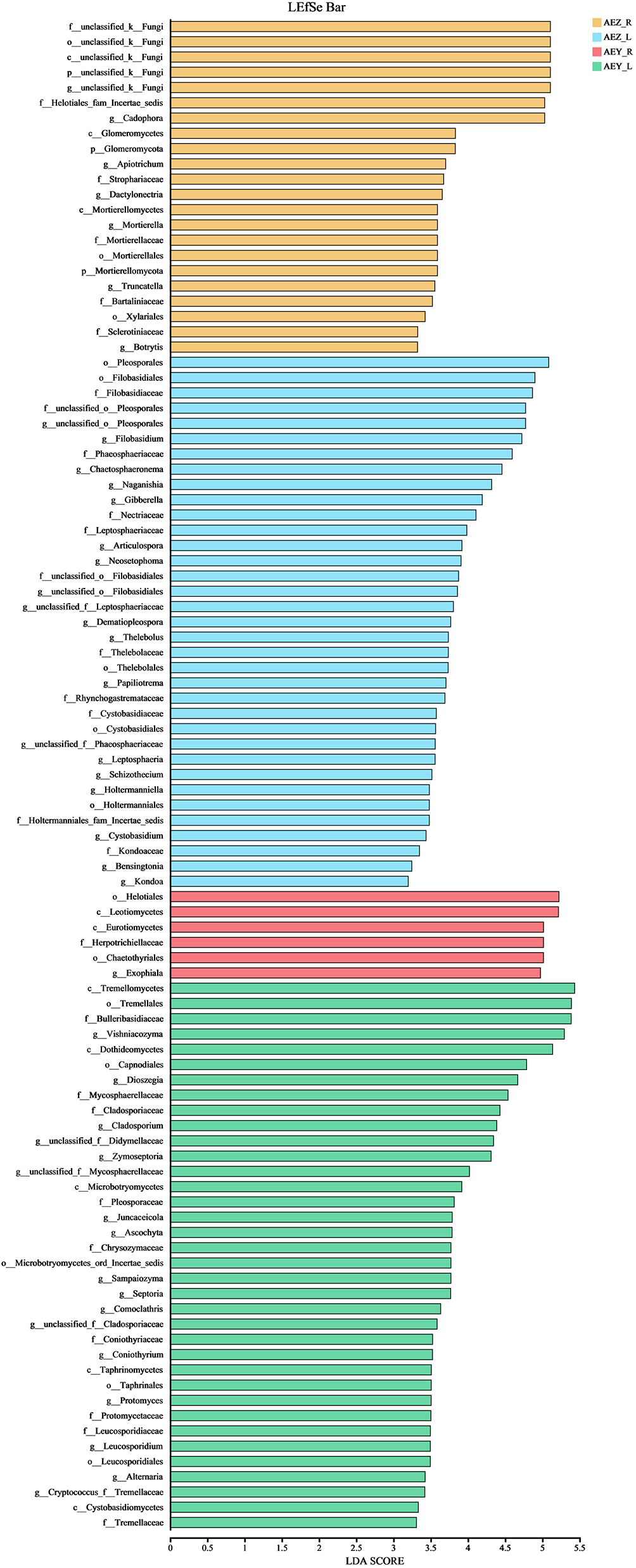

Supplement: Supplementary file 1 [file molecules-30-00734-s001.zip › Figure S1.jpg]

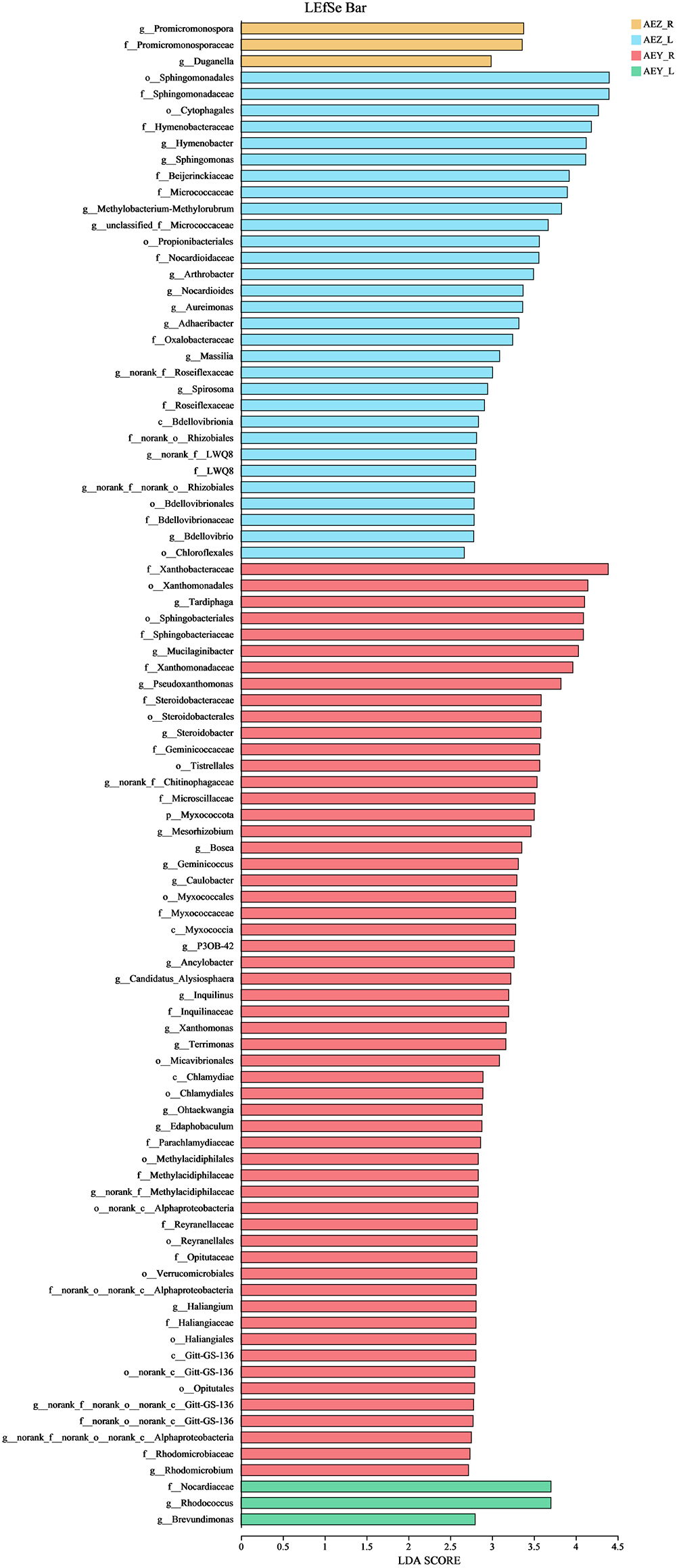

Supplement: Supplementary file 1 [file molecules-30-00734-s001.zip › Figure S2.jpg]

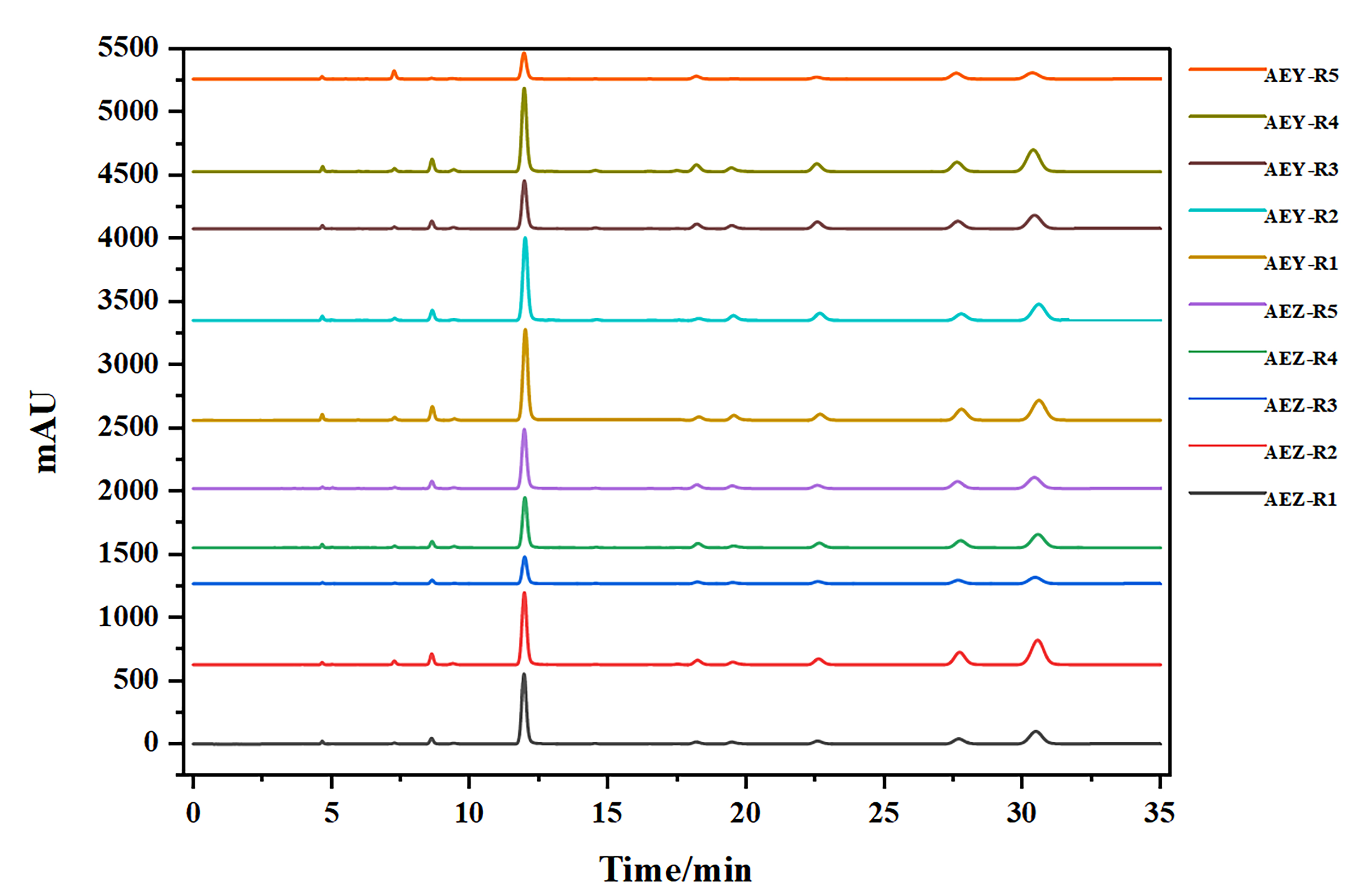

Supplement: Supplementary file 1 [file molecules-30-00734-s001.zip › Figure S3.jpg]
